# Effect of Dry Carbonic Acid Baths on Blood Rheological Parameters in Patients with Venous Leg Ulcers

**DOI:** 10.3390/jcm14186614

**Published:** 2025-09-19

**Authors:** Patrycja Dolibog, Paweł Tomasz Dolibog, Mikołaj Łanocha, Marcelina Paruzel, Tomasz Pryzwan, Aleksandra Frątczak, Wiesław Pilis, Daria Chmielewska, Sławomir Grzegorczyn, Beata Bergler-Czop

**Affiliations:** 1 Department of Medical Biophysics, Faculty of Medical Sciences in Katowice, Medical University of Silesia, 40-055 Katowice, Poland; mparuzel@sum.edu.pl (M.P.); tomasz.pryzwan@sum.edu.pl (T.P.); 2 Department of Biophysics, Faculty of Medical Sciences in Zabrze, Medical University of Silesia, 40-055 Katowice, Poland; pawel.dolibog@sum.edu.pl (P.T.D.); grzegorczyn@sum.edu.pl (S.G.); 3 Department of Dermatology, Faculty of Medical Sciences in Katowice, Medical University of Silesia, 40-055 Katowice, Poland; lanocha.mikolaj@gmail.com (M.Ł.); afratczak@sum.edu.pl (A.F.); bbergler-czop@sum.edu.pl (B.B.-C.); 4 Department of Health Sciences, Collegium Medicum, Jan Długosz University in Częstochowa, 42-200 Częstochowa, Poland; pilwies@wp.pl; 5 Electromyography and Pelvic Floor Muscles Laboratory, Department of Physical Medicine, Institute of Physiotherapy and Health Sciences, The Jerzy Kukuczka Academy of Physical Education, 40-065 Katowice, Poland; d.chmielewska@awf.katowice.pl

**Keywords:** venous ulcers, blood rheology, carbonic acid baths, physical therapy

## Abstract

**Background**: Chronic venous insufficiency (CVI) is one of the main causes of venous leg ulcers. Rheological disorders of the blood, such as changes in viscosity, hematocrit, and erythrocyte aggregation and deformability, can impair microcirculation and impede healing. Carbonic acid (CO_2_) dry baths are a non-invasive physical method that can affect microcirculation and blood parameters, and this study aimed to assess their effectiveness in ulcer healing and in modifying selected blood rheological parameters in patients with CVI. **Methods**: This prospective, controlled study enrolled 23 participants (11 patients with active venous leg ulcers and 12 healthy controls). The intervention group underwent ten sessions of dry CO_2_ baths, performed twice weekly for 5 weeks. No randomization was applied. Ulcer healing was assessed planimetrically, and blood rheological parameters (hematocrit, blood and plasma viscosity, erythrocyte deformability index [EI], aggregation index [AI], aggregation amplitude [AMP], and half-time of aggregation [T_1/2_]) were measured before and after therapy. **Results**: Following the intervention, the ulcer area decreased significantly (median 3.35 cm^2^ to 1.74 cm^2^; *p* < 0.01), as did ulcer circumference (7.33 cm to 5.87 cm; *p* < 0.01). Hematocrit increased (median 40.25% to 41.50%; *p* < 0.05), and blood viscosity values at low shear rates approached those of the control group. In contrast, erythrocyte deformability (EI) and aggregation indices (AI, AMP, T_1/2_) showed no statistically significant intragroup changes, although intergroup differences persisted. Pain intensity decreased significantly (VAS 6.0 to 3.5 cm; *p* < 0.05). **Conclusions**: CO_2_ dry baths support the treatment of venous ulcers by improving microcirculation and reducing pain. Their impact on blood rheology may have clinical significance, especially as an adjunct to therapy in chronic venous insufficiency. However, the relatively small sample size (*n* = 23) should be considered a limitation when interpreting these findings.

## 1. Introduction

Venous leg ulcers (VLUs) are chronic tissue defects resulting from advanced chronic venous insufficiency (CVI). They are a serious clinical, economic and social problem. They occur in 1–3% of adults in developed countries, and their incidence increases with age—up to 5% in people over 65 years of age [1]. Ulcers lead to a decrease in the quality of life of patients, reduced mobility, chronic pain, as well as significant systemic costs associated with long-term treatment and the risk of recurrence [2].

The pathogenesis of venous ulcers is complex and includes venous hypertension, valvular damage, venous reflux, chronic inflammation, tissue hypoxia, and angiogenesis disorders. A particularly important, although often underestimated, role in this context is played by blood rheology—i.e., the science of blood flow properties and its morphotic elements. Parameters such as plasma viscosity, erythrocyte deformability, their aggregation and half-life have a direct impact on the efficiency of microcirculation and oxygen supply to tissues [3,4].

To better understand the therapy’s effects on microcirculation, selected blood rheological parameters were assessed. Blood viscosity, a determinant of flow resistance, may slow capillary flow and induce tissue hypoxia, contributing to ulcer persistence [5,6]. Hematocrit directly affects viscosity and oxygen transport; low values impair oxygenation, whereas high values increase vascular resistance and thrombosis risk [7]. The Elongation Index (EI) reflects red blood cell deformability, with reductions worsening microflow [8]. The Aggregation Index (AI) and Amplitude (AMP) describe erythrocyte clumping that can obstruct microvessels [9]. The half-time of aggregation (T_1/2_) indicates aggregation dynamics, where shortening signals pathological adhesion in chronic venous insufficiency [10]. Rheological abnormalities observed in patients with CVI can lead to deterioration of oxygen transport, increased vascular resistance and chronic skin hypoxia, which promotes the development and fixation of ulcers. In this light, the improvement of rheological parameters may be an important element of therapy supporting the treatment of chronic tissue defects of venous etiology [5].

Scientific research indicates that rheological disorders are not only a secondary phenomenon associated with chronic venous insufficiency, but may also be an independent risk factor for the development of ulcers. Słoczyńska et al. showed that patients with chronic venous insufficiency exhibit significant changes in erythrocyte deformability and aggregation, which affects the deterioration of microcirculation [8]. On the other hand, Monkos et al. They noted that plasma viscosity correlates positively with the severity of trophic changes and delayed healing [6]. In this context, the assessment of selected rheological indicators—including EI, AI, T_1/2_, hematocrit and viscosity—can not only help assess microvascular status, but also provide a tool for monitoring the effectiveness of physical therapies such as dry carbonic acid baths.

Standard treatment for venous leg ulcers includes compression therapy, topical treatment, phlebotropic pharmacotherapy, and in some cases, surgery. However, the effectiveness of these methods is sometimes limited, and the duration of treatment is often long [2,11]. For this reason, supportive therapies are sought—especially those that improve tissue perfusion, reduce inflammation and can affect blood rheology.

One such method is dry carbonic acid baths, which involve the administration of carbon dioxide (CO_2_) in a special gas chamber. CO_2_ penetrates the skin and has a number of physiological effects: it dilates blood vessels (vasodilatory effect), improves tissue oxygenation (Bohr effect), lowers the expression of inflammatory cytokines (Interleukin 6, IL-6; Tumor Necrosis Factor alpha, TNF-α) and stimulates angiogenesis [12,13]. Moreover, there are indications that CO_2_ has an effect on improving blood rheology, including increased erythrocyte elasticity and reduced erythrocyte aggregation [4,14].

The aim of this study was to evaluate the efficacy of carbonic acid dry baths as an adjunct to the treatment of venous leg ulcers. The aim of the study was not only to determine the effect of this form of therapy on the course of ulcer healing, but also to analyze changes in selected blood rheological parameters, such as erythrocyte deformability index (EI), erythrocyte aggregation index (AI), half-life aggregation (T_1/2_), blood viscosity and hematocrit.

In particular, this study investigated whether the use of a CO_2_ dry bath can improve microcirculation by modifying the flow properties of the blood and thus support the process of tissue regeneration in the course of chronic venous insufficiency. This study also investigated whether improved rheological parameters correlated with clinical improvement in venous ulcer health, which could confirm their value as potential biomarkers of therapeutic effectiveness.

## 2. Materials and Methods

**Figure 1:**
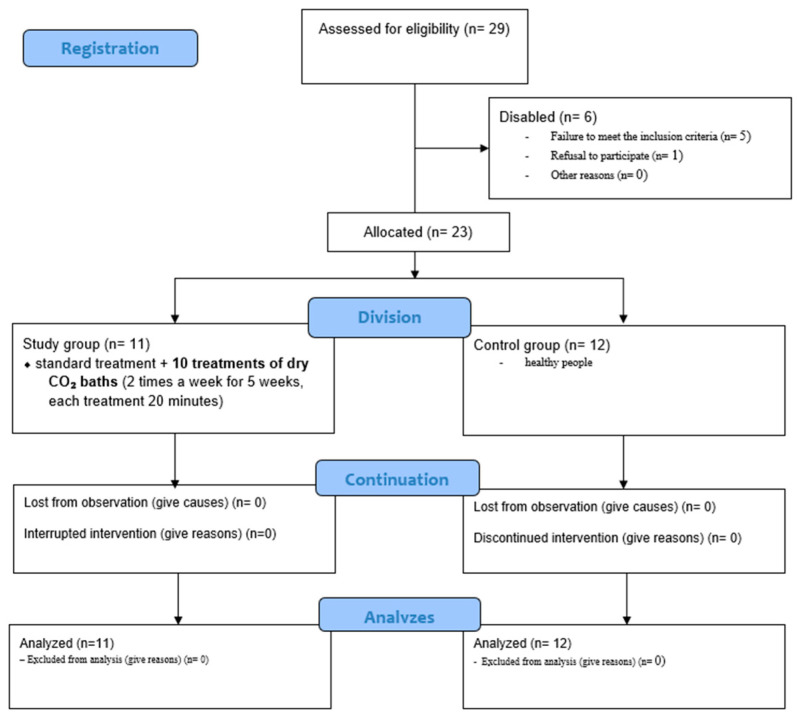
Flow diagram of participant recruitment. allocation. intervention. and analysis in the study.

The study involved 29 patients, of whom 23 were qualified to participate. Six individuals were excluded—five because they did not meet the inclusion criteria and one who withdrew before the intervention (Figure 1). Participants were assigned to two groups: a study group with venous leg ulcers treated with dry carbonic acid baths (*n* = 11) and a control group of healthy volunteers without chronic venous insufficiency (*n* = 12). The control group was matched to the study group in terms of age, gender, and BMI, as presented in Table 1. The study was designed as a prospective, controlled, open-label trial without randomization. To minimize bias, planimetric wound measurements were performed using coded photographs, and laboratory analyses of blood samples were conducted in a blinded manner. The study was carried out between January 2024 and June 2025, with approval from the Bioethics Committee of the Medical University of Silesia in Katowice (BNW/NWN/0052/KB1/30/I/23, approval date: 27 June 2023). All procedures conformed to the principles of the Declaration of Helsinki, and each participant provided written informed consent. The study was prospectively registered in the Australian and New Zealand Clinical Trials Registry (ACTRN12624000636550). All patients underwent Doppler ultrasound examination of the arteries and veins of the lower limbs before starting the procedures, which confirmed the diagnosis of chronic venous insufficiency in accordance with specialist assessment by a dermatologist and a vascular surgeon. Additionally, the ankle–brachial pressure index (ABPI) was measured in each participant, and in all cases it was higher than 0.8.

The study group included adults over 18 years of age with active venous leg ulcers classified as CEAP C6, with an ABPI value above 1.3. Participants had to have the ability to give informed consent and not show contraindications to CO_2_ therapy. Contraindications include active skin infections, severe respiratory and circulatory failure, and intolerance to gas therapy (e.g., dyspnea, deterioration of respiratory comfort). In the study group, the mean age was 70.7 ± 6.5 years, and BMI was 29.0 ± 4.7 kg/m^2^. In the control group, the mean age of the participants was 65.69 ± 6.97 years, and BMI was 28.57 ± 3.56 kg/m^2^, with an ankle–brachial index (ABI) value ranging from 0.8 to 1.3.

**Figure 2:**
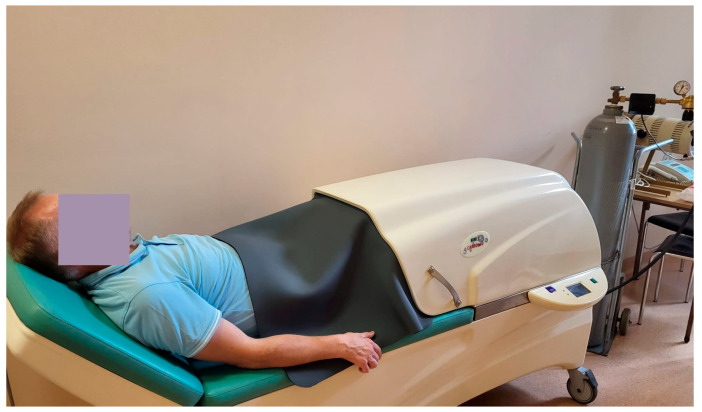
Application of dry carbon dioxide (CO_2_) bath using the CarboBed medical device.

Patients in the study group underwent 10 sessions of dry carbonic acid baths, conducted twice weekly over five weeks, each lasting 20 min. The procedures utilized the CarboBed^®^ medical device (Elektronika I Elektromedycyna Sp.j., Otwock, Poland), CE-certified for clinical CO_2_ therapy in anhydrous conditions [14]. The device features a sealed gas chamber accommodating only the patient’s lower limbs, while the upper body remains outside. Its design allows fully automated treatment delivery, including rapid sealing of the chamber, automatic CO_2_ refill and circulation, and controlled discharge after the session, ensuring high and stable gas concentrations [5,6,7]. The system affords efficient operation (notably lower CO_2_ consumption than wet baths) and enhanced patient comfort due to the ability to perform procedures without disrobing [5,6,7]. Treatment parameters were standardized for all participants: carbon dioxide concentration of 95–100%, temperature maintained at 35 °C (monitored within the device’s adjustable 30–40 °C ±1 °C range), and duration of 20 min. CO_2_ saturation was verified for each session using a Uni-T A37 CO_2_ meter to guarantee consistency and safety. The mobile design, adjustable headrest, and compact dimensions (approx. 215 × 70 × 98 cm; seat height 59 cm) facilitated ease of use (Figure 2) [5,7].

For the planimetric analysis of ulcers, AutoCAD software 2013 (Autodesk, San Francisco, CA, USA) was used to obtain precise and repeatable measurements of wound surface area and perimeter. Photographs of the ulcers were taken with a high-resolution digital camera, positioned perpendicularly (90°) to the wound surface, with a 10 cm calibration ruler placed adjacent to the lesion. Each image was calibrated in AutoCAD, and the wound margins were outlined. Surface area and circumference were then automatically calculated. In this study, all measurements were performed in triplicate by the same trained investigator to minimize intra-observer variability [15]. In order to assess the effect of the therapy on rheological parameters of blood, 5 mL of venous blood was collected from the ulnar vein at rest, in the morning and on an empty stomach, both before and after the start of therapy. Blood samples were protected against permanent changes in blood with an anticoagulant (sodium edetate, K2EDTA). The measurement of blood rheological parameters was carried out using the LORRCA (Laser-assisted Optical Rotational Cell Analyzer, RR Mechatronics, Zwaag, The Netherlands), which allows the assessment of deformability and aggregation of erythrocytes under controlled conditions. Venous blood was collected for heparin and analyzed within two hours of collection, according to the manufacturer’s recommendations [16,17].

To assess the erythrocyte deformability index (EI), a blood sample (12.5 μL) diluted in a polyvinylpyrrolidone (PVP) buffer solution (2.5 mL) was placed (880 µL) in the measurement gap of the analyzer and then subjected to a shear stress gradient. Measurements were performed at a stable temperature of 37 ± 0.2 °C. Changes in erythrocyte shape were monitored using laser ektacytometry by analyzing the elliptical pattern of light scattering. LORCA calculates the EI index based on the ratio of the scattering axes: (L − S)/(L + S) (where L is the major axis of the ellipse and S is the minor axis), as a function of shear stress (0–30 Pa), generating the so-called deformability curve. The EI coefficient reflects the degree of erythrocyte deformation [1,18].

Erythrocyte aggregation was measured using the photometric method. First, 1 mL of whole blood was aerated in a ratio of 1:3 for 5 min (Universal Hematological Mixers, UMH-5, WIGO, Pruszków Poland). A sample of 880 μL of aerated blood was measured in the Lorrca device. The blood was first measured with laser light in laminar flow conditions, after which the flow was stopped. Then, changes in light transmission were recorded, which corresponded to the formation of erythrocyte aggregates. On this basis, the aggregation index (AI) was calculated, which determines the speed and intensity of aggregation, and the half-life (T_1/2_), i.e., the time needed to reach half of the maximum light transmission (formation of 50% of aggregates); the AMP amplitude syllectogram represents the extent of aggregation. The measurement was also carried out in a stabilized temperature of 37 ± 0.2 °C [19,20,21].

Blood viscosity was assessed using the Brookfield DVN rotational viscometer (AMETEK Brookfield, Middleboro, MA, USA), adapted to measure biological liquids under controlled temperature conditions (PolyScience Thermostat, SD07R-20-A12E, Niles, IL, USA). The measurement was performed using a system with CPA-40Z spindle, designed for small volume and low viscosity samples. For each analysis, 0.5 mL of freshly collected, non-hemolyzed whole blood was used, previously kept in a water bath at 37 ± 0.2 °C for 15 min. The measurement was carried out at different spindle speeds, corresponding to changes in shear rate from 15 to 225 s^−1^. In turn, plasma viscosity was measured at two spindle speeds, which corresponded to shear rates of 600 and 900 s^−1^. To obtain pure plasma, whole blood was centrifuged for 5 min at 3000 rpm in a laboratory centrifuge (MFW-251, MPW MED. INSTRUMENTS, Warsaw, Poland). This made it possible to assess both viscosity under laminar flow conditions (low D) and dynamic conditions (high D), which is important for the analysis of blood behavior in different segments of the circulatory system. Viscosity was recorded in mPa·s (millipascal seconds) units, and each sample was analyzed three times. To ensure accuracy and repeatability, the viscometer was calibrated before each measurement in accordance with the manufacturer’s guidelines. In addition, the measurement aperture size was adjusted prior to each whole blood or plasma assay, which resulted in a maximum torque variation of only ±0.2%. All measurements were performed by the same investigator to minimize variability [22,23,24].

The hematocrit value was determined by the microcapillary method, considered to be the standard technique for assessing the ratio of erythrocyte volume to whole blood volume. Blood was collected into heparinized capillaries, which were then sealed with wax and centrifuged in a microcentrifuge (MPW-55, MPW MED. INSTRUMENTS, Poland) for 5 min at 14,000 rpm.

After centrifugation, the length of the erythrocyte column and the total length of the blood column (including plasma and leukocyte layer) were read, and the hematocrit value was expressed as a percentage ratio of erythrocyte volume to total volume (Hct [%]).

The measurement was performed in three replicates for each sample, and the mean value was taken for analysis [25].

Statistical analyses were performed using the Statistica software package version 13.3 (TIBCO Software Inc., San Ramon, CA, USA, 2017). All estimated quantitative variables were verified using the Shapiro–Wilk test to determine the type of distribution. Changes before and after therapy within the study group were assessed using the Wilcoxon signed-rank test for dependent samples. Comparisons between the study and control groups were conducted using the Mann–Whitney U test. In contrast, comparisons of qualitative variables between groups were made using the chi-square test (χ^2^). Spearman’s rank correlation was used to assess the relationships between the examined variables. Because nonparametric tests were applied exclusively (Wilcoxon and Mann–Whitney), no correction for multiple measurements was necessary. A *p*-value of less than 0.05 was considered statistically significant.

## 3. Results

Planimetric analysis showed a significant reduction in the area and circumference of venous ulcers after a series of dry carbonic acid baths. The median ulcer area before treatment was 3.35 cm^2^ (IQR: 2.41–5.45), while after treatment, it decreased to 1.74 cm^2^ (IQR: 0.61–3.13), which was a reduction of nearly 48% (*p* = 0.0022; Wilcoxon test). The circumference of trophic lesions was also significantly reduced—from 7.33 cm (IQR: 6.23–10.91) to 5.87 cm (IQR: 4.38–7.88) (*p* = 0.0029). Detailed data are presented in Table 2.

In terms of rheological parameters, an increase in hematocrit values was observed in the study group—before therapy the median was 40.25% (IQR: 35.50–42.00), and after therapy 41.50% (IQR: 37.00–45.00). In the control group that did not receive the intervention, the median hematocrit was 45.00% (IQR: 43.00–47.00). The differences between the study and control groups were statistically significant both before and after the start of therapy (*p* = 0.0003) (*p* = 0.0422), as shown in Table 3. These baseline disparities may complicate the interpretation of post-treatment changes, since the groups were not fully comparable at the outset.

In terms of rheological properties of blood, a characteristic, non-linear course of blood viscosity values was observed at different shear velocities (D [s^−1^]). After therapy, the viscosity values in the study group approached the levels observed in the control group, especially in the lower velocity range (D = 15, 30 and 60 s^−1^). Although the changes within the study group did not reach the level of statistical significance (*p* > 0.05), significant differences were noted between the pre-treatment and control groups at several measurement points: for D = 15 (*p* = 0.0086), D = 30 (*p* = 0.0055), D = 60 (*p* = 0.0012) and D = 112.5 s^−1^ (*p* = 0.0034). This may indicate a tendency to normalize hematological parameters after therapy.

The values of erythrocyte aggregation indices showed significant differences between the study and control groups. Aggregation amplitude (AMP) and aggregation index (AI) were significantly higher in patients both before and after treatment compared to controls (AMP: *p* = 0.0220 and 0.0101, AI: *p* = 0.0350 and 0.0021), with no significant changes within the study group. The half-life (T_1/2_) after therapy was significantly shorter than in the control group (*p* = 0.0101), which may suggest an increased reactivity of the red blood cell system to a physical stimulus. Detailed data are presented in Table 4.

The deformability index of erythrocytes (EI) was evaluated in the range of different shear stresses (0.3–30 Pa). In the analysis, no significant changes were observed within the post-therapy group (*p* > 0.05), and the index values remained stable and showed a similar course both before and after the intervention. Comparison of the study and control groups revealed only one significant difference—the EI value at 30 Pa stress, where the index was lower in the pre-therapy group (*p* = 0.0473). After the end of therapy, this difference ceased to be statistically significant, which may suggest partial normalization of this parameter. Detailed results are presented in Table 5.

The analysis showed a statistically significant reduction in pain intensity after ulcer treatment. The mean decrease in VAS scores was 38.77 ± 34.42%, and the median was 25.40%, indicating significant clinical improvement. The interquartile range was 11.46 (Q1) to 66.67 (Q3). The test showed that the lesion was statistically significant (*p* = 0.012), which confirms the effectiveness of the treatment in reducing pain.

**Figure 3:**
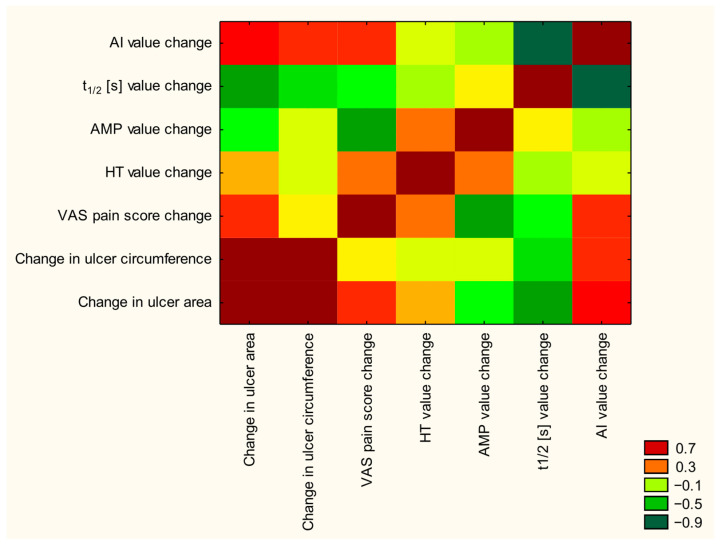
Spearman correlation matrix showing relationships between clinical and hemorheological parameters in patients undergoing CO_2_ dry bath therapy. Significant negative correlations were observed between ulcer area reduction and T_1/2_ (r = −0.64). AMP and VAS pain score (r = −0.66). as well as between AI and T_1/2_ (r = −0.94). The color scale reflects the strength and direction of correlation (from −0.9 to +0.7).

Spearman rank correlation analysis showed a statistically significant positive relationship between the change in ulcer surface area and the change in ulcer circumference (r = 0.90; *p* < 0.001), suggesting consistency between the two parameters in the assessment of healing progression. In addition, a significant moderate negative correlation was found between the change in ulcer surface area and the time T_1/2_ (r = −0.64; *p* = 0.048), which may indicate a relationship between wound shrinkage and improvement of microvascular parameters. Amplitude values (AMP) showed a significant, strong negative correlation with the change in pain perception on the VAS scale (r = −0.66; *p* = 0.036), which may suggest that better microvascular parameters are associated with pain reduction. A very strong, negative and significant correlation was also noted between the time T_1/2_ and the AI index (r = −0.94; *p* < 0.001), which may indicate a correlation of hemodynamic changes in the assessed area. The remaining correlations, although in some cases showed moderate strength (e.g., AI vs. ulcer area: r = 0.48; *p* = 0.16), did not reach the level of statistical significance (*p* > 0.05), which may be due to a limited number of observations or the co-occurrence of other confounders (Figure 3).

## 4. Discussion

The results of the study indicate that dry carbonic acid baths may be an effective form of therapy to support the treatment of chronic venous ulcers. A significant reduction in ulcer area and circumference observed after a series of treatments suggests a beneficial effect of CO_2_ on tissue regeneration processes. These effects confirm the previous observations of Prazeres [12], who showed that CO_2_ baths improve tissue perfusion and oxygenation and reduce the expression of pro-inflammatory cytokines.

Although changes in blood rheological parameters did not reach statistical significance in most cases, a clear tendency to normalize them was observed, especially with regard to hematocrit and blood viscosity. These trends should be interpreted with caution, as statistical significance was not consistently achieved. The hematocrit value in the study group increased after therapy from a median of 38.85% to 41.10%, which indicates an improvement in the oxygen transport potential while maintaining safe physiological limits. On the other hand, blood viscosity, although it did not significantly decrease, after the therapy approached the values observed in the control group, which may indicate an improvement in the fluidity of blood flow in microcirculation. The effect of dry CO_2_ in this respect may be associated with vasodilation and improved vascular elasticity. In addition, a reduction in oxidative stress could influence plasma properties and the composition of blood morphotic elements [5,12,14]. In addition, research by Prazeres and Zbroja [12,14] has shown that CO_2_ baths can improve flow in the vessels of the lower limbs by affecting rheology and improving cell metabolism. Therefore, the observed changes in hematocrit and viscosity may be a consequence not only of improved perfusion, but also of the beneficial effects of CO_2_ on the overall hemodynamic status of patients.

The lack of significant changes in erythrocyte deformability and aggregation (EI, AI, AMP, T_1/2_) may be due to the limited duration of therapy (10 treatments, 5 weeks) or the stage of the disease in patients. Literature data suggest that rheological effects may appear only after prolonged exposure or in combination with other forms of treatment [4,6,14]. Nevertheless, the observed trends in rheological parameters, combined with clinical improvement and reduction in patient pain (VAS), provide the basis for further research in this area.

It is also worth noting that some parameters, such as half-life aggregation (T_1/2_), showed intergroup differences after therapy, which may indicate increased reactivity of the red blood cell system to a physical stimulus. The potential of CO_2_ dry baths as a non-invasive method to support the treatment of venous ulcers, especially in the context of blood rheology disorders, is promising and deserves further exploration in studies with larger numbers of participants and extended follow-up time. Ideally, such trials should be multicenter to increase generalizability.

In the context of current therapeutic standards, which often do not bring the expected results in patients with a long history of CVI, the inclusion of balneotherapy with the use of dry CO_2_ can be a valuable complement to the comprehensive procedure. An additional advantage of this method is its safety, low invasiveness and high acceptability by patients.

The results so far encourage further research into the long-term efficacy of the therapy and the possibility of combining CO_2_ with other treatments—both pharmacological and physical.

It is worth noting that the effectiveness of carbonic acid dry baths may be related to the multifactorial mechanism of action. In addition to the aforementioned vasodilation effect, an important aspect is also the improvement of metabolic conditions within the tissues affected by trophic changes. Research by Macura et al. [13] showed that transdermal application of CO_2_ leads to the activation of vascular endothelial growth factor (VEGF), which supports neovascularization processes. In addition, Prazeres and Zbroja [12,14] suggest that CO_2_ baths may improve capillary flow not only through the Bohr effect, but also by reducing oxidative stress and improving cell mitochondrial function.

Comparing our results with the studies by Dolibog et al. [1], who evaluated the effectiveness of shockwave therapy and compression therapy in the treatment of VLU, it can be noted that a dry carbonic acid bath, despite its lower technological advancement, can achieve a similar clinical effect with much less financial outlay and greater patient comfort. In this context, this method can be used not only in outpatient conditions, but also in home care of chronically ill patients.

This study has several limitations. The small sample size may reduce statistical power and obscure relevant effects, particularly in rheological parameters where trends did not reach significance. Moreover, the lack of randomization and blinding could influence subjective outcomes like pain intensity (VAS). Finally, the short follow-up period limits conclusions on long-term efficacy. Additionally, the single-center design may introduce selection bias, and baseline differences in hematocrit values could act as potential confounding factors. Future studies with larger cohorts, extended observation, and inclusion of inflammatory markers (e.g., TNF-α, VEGF) are warranted.

In addition, the correlation analysis confirmed significant relationships between clinical and rheological parameters, which may indicate the importance of microcirculation in the process of venous wound healing. A moderate, significant negative correlation was observed between the change in ulcer surface area and the time T_1/2_ (r = −0.64; *p* = 0.048), suggesting that improved aggregation properties of erythrocytes may promote tissue regeneration. Also, a strong, significant negative correlation between the AMP value and the reduction of pain (r = −0.66; *p* = 0.036) indicates a possible relationship between hemodynamic parameters and subjective improvement experienced by patients. In addition, a very strong, significant negative correlation between T_1/2_ and the AI index (r = −0.94; *p* < 0.001) confirms the internal consistency of the evaluated rheological indicators.

These results may suggest the potential usefulness of selected rheological parameters as biomarkers of response to physical therapy. However, this should be regarded as a hypothesis that requires confirmation in larger, prospective studies. Although not all of the analyzed relationships reached the level of statistical significance, their direction and strength indicate the legitimacy of further studies with a larger group of patients.

Taken together, the results provide a strong starting point for further research on gas balneotherapy as an adjunctive element in the treatment of chronic venous ulcers. Combining this method with the assessment of blood rheology provides a valuable tool for assessing the effectiveness of the intervention and its impact on microvascular physiology.

## 5. Conclusions

Dry carbonic acid baths lead to a significant reduction in the area of venous leg ulcers, which indicates their effectiveness as a method of supporting treatment. CO_2_ therapy is safe, well tolerated by patients and does not cause side effects, making it an attractive option for outpatient treatment.

CO_2_ dry bath therapy may promote beneficial changes in selected hemodynamic parameters, in particular hematocrit and blood viscosity at low shear rates. However, these findings should be interpreted with caution, as several rheological indices did not reach statistical significance. The observed trends may suggest possible support of physiological autoregulation of the cardiovascular system.

Chronic venous insufficiency is associated with permanent disturbances in the rheological properties of blood, which are not significantly modulated as a result of short-term physical therapy. The observed correlations suggest that selected blood indices may reflect the effects of therapy and should be taken into account in the assessment of its effectiveness.

## Figures and Tables

**Table 1:** Baseline characteristics of the study and control groups. Data are presented as mean ± SD, median, and interquartile range (Q1–Q3). *p*-values were calculated using the Mann–Whitney U test; * *p*-value for gender distribution based on χ ^2^ test.

Variable	Study Group (*n* = 11)					Control Group (*n* = 12)					*p*-Value
	Mean	SD	Median	Q1	Q3	Mean	SD	Median	Q1	Q3	
Age [years]	70.70	6.50	72.50	65.00	77.00	65.69	6.97	67.00	60.00	71.00	0.0883
BMI [kg/m^2^]	28.98	4.68	29.07	28.01	33.05	28.57	3.56	28.00	25.50	32.00	0.4833
GENDER *n*	F = 3 M = 8					F = 2 M = 10					0.5379 *

**Table 2:** Comparison of wound size (area and perimeter) before and after the intervention in the study group. Data are presented as mean ± SD, median and interquartile range (Q1–Q3). Statistically significant differences (*p* < 0.05) were observed using the Wilcoxon signed-rank test.

Variable	Initial Wound					Final Wound					*p*-Value
	Mean	SD	Median	Q1	Q3	Mean	SD	Median	Q1	Q3	
size area [cm^2^]	7.45	11.77	3.35	2.41	5.45	2.85	3.79	1.74	0.61	3.13	0.0022
size perimeter [cm]	10.49	9.58	7.33	6.23	10.91	6.41	3.64	5.87	4.38	7.88	0.0029

**Table 3:** Hematocrit values (%) in the study and control groups before and after the intervention. Data are presented as mean ± SD, median and interquartile range (Q1–Q3). Statistically significant differences were observed between groups both before and after the intervention (*p* < 0.05; Mann–Whitney U test).

Variable	Study Group (*n* = 11)					Control Group (*n* = 12)					*p*-Value
	Mean	SD	Median	Q1	Q3	Mean	SD	Median	Q1	Q3	
HT before [%]	38.85	5.15	40.25	35.50	42.00	45.23	2.41	45.00	43.00	47.00	0.0003
HT after [%]	41.10	4.53	41.50	37.00	45.00						0.0422

**Table 4:** Erythrocyte aggregation indices and blood and plasma viscosity [mPa·s] at various shear rates (D [s^−1^]) in the study and control groups. Data are presented as mean ± SD, median and interquartile range. Statistically significant differences (*p* < 0.05). Comparisons were made using the Wilcoxon signed-rank test (pre vs. post) and the Mann–Whitney U test (between groups).

Parameters Mean ± SD Median (Q1–Q3)	Study Group (pre)	Study Group (post)	Control Group	*p* (Study Group Pre vs. Post)	*p* (Study Group Pre vs. Control)	*p* (Study Group Post vs. Control)
AMP [au]	19.28 ± 3.8 19.8 (15.2–22.9)	17.75 ± 5.7 18.05 (14.5–21.71)	23.93 ± 3.53 25.7 (22.21–26.1)	0.2845	0.0220	0.0101
AI [%]	70.29 ± 7.36 73.71 (61.22–74.77)	73.2 ± 6.36 75.52 (66.66–77.08)	61.9 ± 6.83 62.54 (61.03–65.89)	0.2845	0.0350	0.0021
T_1/2_ [s]	1.63 ± 0.69 1.28 (1.14–2.4)	1.4 ± 0.54 1.17 (1–1.84)	2.4 ± 0.86 2.31 (1.82–2.38)	0.0745	0.0653	0.0101
Blood viscosity [mPas] (15)	7.83 ± 1.9 7.66 (6.59–9.2)	9.37 ± 1.95 9.66 (8.05–11.04)	11.35 ± 4.16 9.89 (9.5–12.42)	0.0747	0.0086	0.4411
Blood viscosity [mPas] (30)	5.62 ± 1.38 5.67 (4.75–5.98)	6.5 ± 1.4 6.02 (5.52–7.89)	8.19 ± 2.57 7.78 (6.67–8.66)	0.1282	0.0055	0.0698
Blood viscosity [mPas] (60)	4.83 ± 0.92 4.68 (4.33–5.25)	5.61 ± 1.08 5.27 (4.78–6.54)	6.9 ± 1.94 6.53 (5.36–7.13)	0.1763	0.0012	0.4411
Blood viscosity [mPas] (112.5)	4.33 ± 0.74 4.37 (3.92–4.84)	4.85 ± 0.75 4.59 (4.24–5.49)	5.63 ± 1.15 5.42 (4.66–6.03)	0.1159	0.0034	0.0950
Blood viscosity [mPas] (225)	3.89 ± 0.65 3.95 (3.63–4.11)	4.34 ± 0.57 4.29 (3.82–4.7)	5 ± 0.93 5.05 (4.15–5.42)	0.1282	0.0027	0.0698
Plasma viscosity [mPas] (600)	1.77 ± 0.37 1.67 (1.62–1.82)	1.79 ± 0.14 1.77 (1.69–1.85)	1.65 ± 0.18 1.64 (1.53–1.78)	0.3105	0.5027	0.0908
Plasma viscosity [mPas] (900)	1.68 ± 0.26 1.58 (1.57–1.74)	1.69 ± 0.13 1.66 (1.59–1.74)	1.55 ± 0.12 1.56 (1.46–1.67)	0.3105	0.3312	0.0620

**Table 5:** Erythrocyte deformability index (EI) at increasing shear stress (Pa) in the study and control groups. Data are shown as mean ± SD, median and interquartile range. Statistically significant differences (*p* < 0.05). Comparisons were made using the Wilcoxon signed-rank test and Mann–Whitney U test.

Parameters Mean ± SD Median (Q1–Q3)	Study Group (pre)	Study Group (post)	Control Group	*p* (Study Group pre vs. post)	*p* (Study Group pre vs. Control)	*p* (Study Group post vs. Control)
EI 0.3 [Pa]	0.1 ± 0.02 0.11 (0.09–0.11)	0.11 ± 0.02 0.11 (0.09–0.12)	0.09 ± 0.02 0.1 (0.08–0.1)	0.4446	0.3316	0.1164
EI 0.53 [Pa]	0.17 ± 0.02 0.17 (0.15–0.19)	0.17 ± 0.02 0.17 (0.16–0.19)	0.15 ± 0.02 0.15 (0.13–0.17)	1.0000	0.2169	0.1164
EI 0.95 [Pa]	0.25 ± 0.02 0.25 (0.24–0.27)	0.25 ± 0.02 0.24 (0.23–0.27)	0.23 ± 0.02 0.24 (0.21–0.25)	0.6891	0.1164	0.2426
EI 1.69 [Pa]	0.34 ± 0.02 0.33 (0.32–0.36)	0.34 ± 0.02 0.33 (0.32–0.35)	0.32 ± 0.02 0.33 (0.31–0.34)	0.7221	0.3316	0.2703
EI 3 [Pa]	0.4 ± 0.02 0.4 (0.39–0.43)	0.41 ± 0.02 0.4 (0.39–0.41)	0.39 ± 0.01 0.4 (0.39–0.4)	0.6566	0.4385	0.3653
EI 5.33 [Pa]	0.47 ± 0.02 0.47 (0.46–0.49)	0.47 ± 0.02 0.47 (0.46–0.48)	0.47 ± 0.01 0.47 (0.46–0.47)	0.8589	0.8977	0.7969
EI 9.49 [Pa]	0.52 ± 0.02 0.52 (0.51–0.53)	0.52 ± 0.01 0.52 (0.51–0.53)	0.52 ± 0.01 0.52 (0.51–0.53)	0.6566	0.6522	0.8977
EI 16.87 [Pa]	0.56 ± 0.01 0.55 (0.55–0.57)	0.56 ± 0.01 0.56 (0.55–0.57)	0.56 ± 0.01 0.56 (0.56–0.57)	0.3505	0.2703	0.4385
EI 30 [Pa]	0.59 ± 0.01 0.59 (0.58–0.6)	0.59 ± 0.01 0.59 (0.59–0.6)	0.6 ± 0.01 0.6 (0.59–0.6)	0.1925	0.0473	0.1164

## Data Availability

The data presented in this study are available on request from the corresponding author.
